# Autophagy Induction by a Small Molecule Inhibits *Salmonella* Survival in Macrophages and Mice

**DOI:** 10.1128/AAC.01536-19

**Published:** 2019-11-21

**Authors:** Toni A. Nagy, Joaquin L. J. Quintana, Abigail L. Reens, Amy L. Crooks, Corrella S. Detweiler

**Affiliations:** a Department of Molecular, Cellular and Developmental Biology, University of Colorado, Boulder, Colorado, USA

**Keywords:** *Salmonella*, chemical genetics, autophagy

## Abstract

Salmonella enterica is a natural bacterial pathogen of humans and animals that causes systemic infection or gastroenteritis. During systemic infection, *Salmonella* generally resides within professional phagocytes, typically macrophages, whereas gastroenteritis is caused by infection of epithelial cells. We are only beginning to understand which host pathways contribute to *Salmonella* survival in particular cell types. We therefore sought to identify compounds that perturb *Salmonella*-host interactions using a chemical genetics approach. We found one small molecule, D61, that reduces *Salmonella* load in cell line and primary macrophages but has no effect on *Salmonella* growth in epithelial cells or rich medium. We determined that in macrophages, D61 induces LC3II, a marker of the autophagy pathway, and promotes aggregation of LC3II near *Salmonella*. We found that D61 antibacterial activity depends on the VPS34 complex and on ATG5. D61 also reduced *Salmonella* load in the spleens and livers of infected mice. Lastly, we demonstrate that D61 antibacterial activity in macrophages is synergistic with the antibiotic chloramphenicol but that this synergy is largely independent of the known autophagy-stimulating activity of chloramphenicol. Thus, a small molecule has antibacterial activity specifically in macrophages and mice based on the promotion of bacterial degradation by autophagy. These observations demonstrate the potential therapeutic utility of stimulating autophagy in cells and animals to curb infection.

## Abstract (precis)

Salmonella enterica is a natural bacterial pathogen of humans and animals that causes systemic infection or gastroenteritis. During systemic infection, *Salmonella* generally resides within professional phagocytes, typically macrophages, whereas gastroenteritis is caused by infection of epithelial cells. We are only beginning to understand which host pathways contribute to *Salmonella* survival in particular cell types.

## INTRODUCTION

Persistent, clinically relevant human pathogens that live within macrophages include species of *Salmonella*, *Mycobacterium*, and *Brucella*, as well as fungi such as *Cryptococcus* and *Histoplasma*. Elucidating the precise molecular interactions between a pathogen and the host is crucial to understanding infection processes and to developing therapeutics. Salmonella enterica serovar Typhimurium (*Salmonella*) is an excellent model Gram-negative pathogen because it is easy to work with and causes natural infections of humans and mice. *Salmonella* causes gastroenteritis in humans by colonizing epithelial cells in the gut (1). In contrast, prior to systemic infection of mice, *Salmonella* traverses the gut epithelial cell layer and is phagocytosed by cells of the monocyte lineaging, including macrophages, which deliver the pathogen to the spleen and liver (2). Although *Salmonella* has a hyperreplicative phase within the cytosol of epithelial cells, the bacteria are contained in all encountered cell types for at least some time within a specialized vesicular compartment termed the *Salmonella*-containing vacuole (SCV) (3, 4).

To identify small molecules that interfere with *Salmonella* survival or replication in macrophages, we recently screened the 14,400-compound Maybridge HitFinder v11 library in a high-content Screen for Anti-Infectives using Fluorescence microscopy of IntracellulaR *Enterobacteriaceae* (SAFIRE) (5). Infected macrophage-like cells were treated with compound from 2 to 18 h postinfection, and then the cellular load of green fluorescent protein (GFP)-expressing *Salmonella* was quantified using automated imaging. A secondary screen in which lysed macrophages were plated for CFU identified 58 small molecules that reduce *Salmonella* growth and/or survival in macrophages. Unlike traditional antibiotics, these hit compounds lack antibacterial activity in standard microbiological medium (5). Here, we focused on one, D61, which was particularly effective at reducing bacterial load and survival in macrophages.

## RESULTS

### A compound that reduces *Salmonella* load in macrophages.

**FIG 1:**
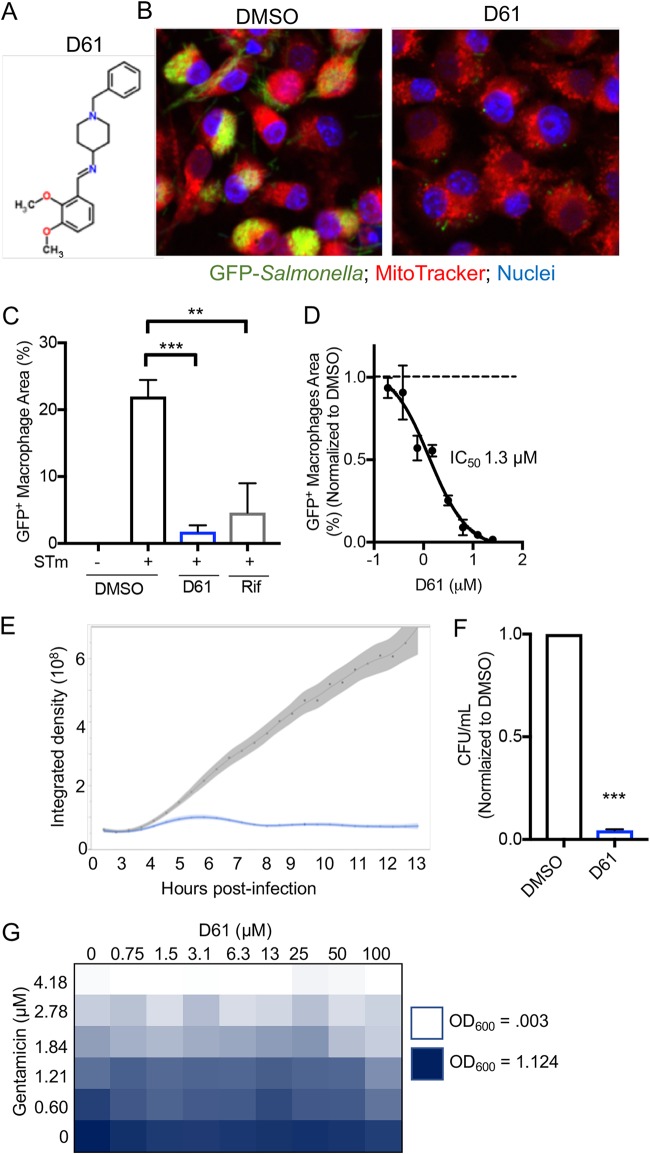
D61 is a potent antibacterial compound in cell culture macrophages. (A) Structure of D61. (B to F) RAW 264.7 macrophage-like cells were infected with stationary-phase *Salmonella* harboring a chromosomal *sifB*::GFP insertion. Cells were treated at 2 h postinfection with vehicle (DMSO), D61, or as indicated. Cells were fixed and imaged (B to D) or lysed and plated (F) to enumerate CFU at 18 h postinfection. (E) Alternatively, cells were live imaged. (B) Representative micrographs from cells treated with DMSO (left) or D61 (25 μM, right). (C) GFP^+^ macrophage area quantified from micrographs of cells treated with DMSO, D61 (25 μM), or rifampin (Rif; 10 μg/ml). The GFP^+^ macrophage area is defined as the number of GFP^+^ pixels per macrophage divided by the total number of pixels per macrophage, averaged across all macrophages. **, *P* ≤ 0.005; ***, *P* ≤ 0.0005 (compared to infected DMSO-treated samples by one-way analysis of variance [ANOVA] with Dunnett’s multiple-comparison test). (D) GFP^+^ macrophage area dose response using 2-fold dilutions of D61 from 25 μM. (E) Live imaging of macrophages over 13 h with DMSO (gray) or D61 (10 μM, blue). The integrated density of GFP^+^ macrophage area across four fields was determined. (F) CFU of cells treated with DMSO or D61 (25 μM). The mean CFU/ml (DMSO) = 8.1 × 10^6^; the mean CFU/ml (D61) = 3.6 × 10^5^. The mean and standard errors of the mean (SEM) of three biological replicates are shown. ***, *P* ≤ 0.0005 (versus DMSO as determined by unpaired *t* test). (G) Wild-type stationary-phase *Salmonella* was grown in MHB with a dose range of D61 and gentamicin. The means and the SEM of two independent biological replicates are shown.

D61 is a small aromatic molecule not previously described as having biological activity (Fig. 1A). In the SAFIRE assay, D61 (25 μM) reduced the bacterial load (GFP signal in RAW 264.7 macrophages) by ∼20-fold, with a half-maximum inhibitory concentration (IC_50_) of 1.3 μM (Fig. 1B to D). Live imaging of RAW 264.7 cells treated with D61 (10 μM) 2 h after infection revealed reduced bacterial GFP signal compared to control within 4 h of compound treatment, suggesting D61 acts rapidly to curb infection (Fig. 1E; see also Movie S1 (dimethyl sulfoxide [DMSO]) and Movie S2 (D61) in the supplemental material). Treatment with D61 (25 μM) also reduced the number of recoverable bacteria in macrophages by 20-fold, as monitored by plating for CFU. Thus, D61 does not simply prevent GFP production but indeed leads to bacterial death within macrophages (Fig. 1F). The antibacterial activity of D61 appears to be specific for intracellular bacteria, since the MIC of this compound in broth is >200 μM. Finally, D61 does not appear to have synergistic activity in broth with gentamicin, the antibiotic used in the cell culture infection experiments to limit extracellular bacterial replication (Fig. 1G). These data indicate that treatment with D61 specifically inhibits *Salmonella* replication and/or survival within cell culture macrophages.

### D61 has antibacterial activity in primary mouse and human macrophages.

**FIG 2:**
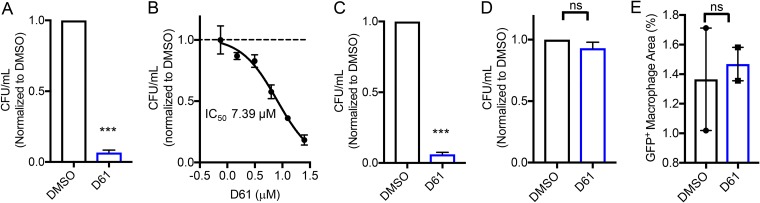
D61 is effective in primary mouse and human macrophages but not in HeLa cells. Mouse primary Sv129 BMDMs (A and B) or human primary BMDMs (C) were infected with wild-type stationary-phase *Salmonella*. Cells were treated at 2 h postinfection with DMSO or D61 (25 μM), and at 18 h postinfection the cells were lysed and plated to enumerate the CFU. The mean CFU/ml (DMSO) in mouse primary macrophages = 5.0 × 10^6^; the mean CFU/ml (D61) in mouse primary macrophages = 2.4 × 10^5^; the mean CFU/ml (DMSO) in human primary macrophages = 1.3 × 10^3^; the mean CFU/ml (D61) in human primary macrophages = 8.9 × 10^1^. (B) Dose response of BMDMs using 2-fold dilutions of D61 from 25 μM. (D) HeLa cells were infected with wild-type stationary-phase *Salmonella*. Cells were treated at 2 h postinfection with D61 (25 μM), and at 18 h postinfection the cells were plated to enumerate the CFU. The mean CFU/ml (DMSO) in HeLa cells = 1.2 × 10^6^; the mean CFU/ml (D61) in HeLa cells = 1.1 × 10^6^. (E) HeLa cells were infected with wild-type stationary-phase *Salmonella* harboring *prpsM*::GFP. Cells were treated at 2 h postinfection with D61 (25 μM), and at 18 h the cells were processed according to the SAFIRE protocol. The data are means and the SEM of four (C and D), three (A and B), or two (E) independent biological replicates. ***, *P* ≤ 0.0005 (relative to DMSO as determined by an unpaired *t* test); ns, not significant.

The RAW 264.7 cell line used in the SAFIRE and CFU assays is a proxy for macrophages and is considerably more permissive for *Salmonella* than resting primary mouse macrophages, due in part to differential production of reactive oxygen and nitrogen species (6–8). RAW 264.7 cells also have homozygous loss-of-function mutations in Nramp1, which encodes an iron transporter that restricts the replication of intravesicular pathogens, including *Salmonella* (8). We therefore examined whether D61 prevents *Salmonella* replication in bone marrow-derived mouse macrophages (BMDMs) from Sv129S6/SvEvTac (Sv129) mice, which are Nramp1^+/+^. Treatment with D61 (25 μM) reduced recovery of bacterial CFU from primary mouse macrophages by 17-fold (Fig. 2A), and the compound has an IC_50_ of 7.9 μM (Fig. 2B). To examine whether D61 is antibacterial in animal species other than mice, we infected primary human bone marrow macrophages with wild-type (WT) *Salmonella* and treated with D61 (25 μM). Treatment reduced recovery of bacterial CFU by 23-fold at 18 h postinfection (Fig. 2C). These data demonstrate that D61 is antibacterial in mouse and human primary macrophages, indicating the compound may have *in vivo* relevance.

### D61 is not antibacterial in HeLa cells and does not impact host cell survival.

While *Salmonella* appears to reside in myeloid cells such as macrophages during systemic infection, gastrointestinal distress is associated with colonization of epithelial cells (9). We therefore examined the effect of D61 on *Salmonella* in HeLa cells, which are derived from the human epithelium. Neither quantification of bacterial load in the SAFIRE assay nor plating lysed HeLa cells for CFU demonstrated that treatment with D61 (25 μM) reduced bacterial load within 18 h of infection (Fig. 2D and E). These experiments, and the macrophage experiments above, were carried out with *Salmonella* grown to stationary phase, which prevents T3SS-1-mediated killing of macrophages by repressing type 3 secretion system 1 (T3SS-1) (10–12). We next infected HeLa cells with *Salmonella* grown to late log phase, which express T3SS-1, but we did not observe D61 antibacterial activity (see Fig. S1 in the supplemental material). Thus, while D61 has antibacterial activity in human macrophages, it does not appear to be similarly active in HeLa cells.

Since host cell death over the course of infection has the potential to confound experimental interpretation of bacterial load (4), we established whether D61 treatment differentially kills RAW 264.7 and/or HeLa cells. First, examination of micrographs of D61 (25 μM) versus DMSO-treated macrophages at 18 h after infection did not suggest differences in cell death (see Fig. S2A in the supplemental material). We then monitored the escape of lactate dehydrogenase (LDH) from uninfected and infected cells with or without D61 treatment. Infection alone enhanced LDH release, as expected. However, treatment with D61 (25 μM) did not significantly increase LDH release compared to treatment with DMSO in either cell type (Fig. S2B and C). We also monitored LDH release across a dosage range of D61 in human HepG2 hepatocytes, which are typically used in toxicity studies. D61 had a half-maximal cytotoxic concentration (CC_50_) of 113 ± 18 μM, which is ∼100-fold higher than the IC_50_ for reducing bacterial load in macrophages (Fig. S2D). Together, these data suggest that differences in D61 activity between macrophages and HeLa cells do not reflect differences in host cell killing.

### The in-cell antibacterial activity of D61 is independent of NOS.

In the SAFIRE protocol, compound is added to host cells 2 h after infection. After 4 h, *Salmonella* numbers in RAW 264.7 cells diverge from the DMSO treatment controls (Fig. 1E). Therefore, D61 may amplify or extend early macrophage defenses, including the production of reactive nitrogen species (NOS) (7, 13, 14). We measured supernatant NOS using a Griess assay. Treatment with D61 had no or modest effects on NOS accumulation whether cells were infected or not (Fig. S3A). As expected, treatment with the iNOS inhibitor L-nil reduced nitric oxide accumulation (Fig. S3A). However, neither L-nil nor a scavenger of H_2_O_2_-derived oxidants (acetovanillone) altered the antibacterial activity of D61 (Fig. S3B). Thus, D61 antibacterial activity may be independent of NOS.

### *Salmonella* is suppressed by D61 over the course of macrophage infection.

**FIG 3:**
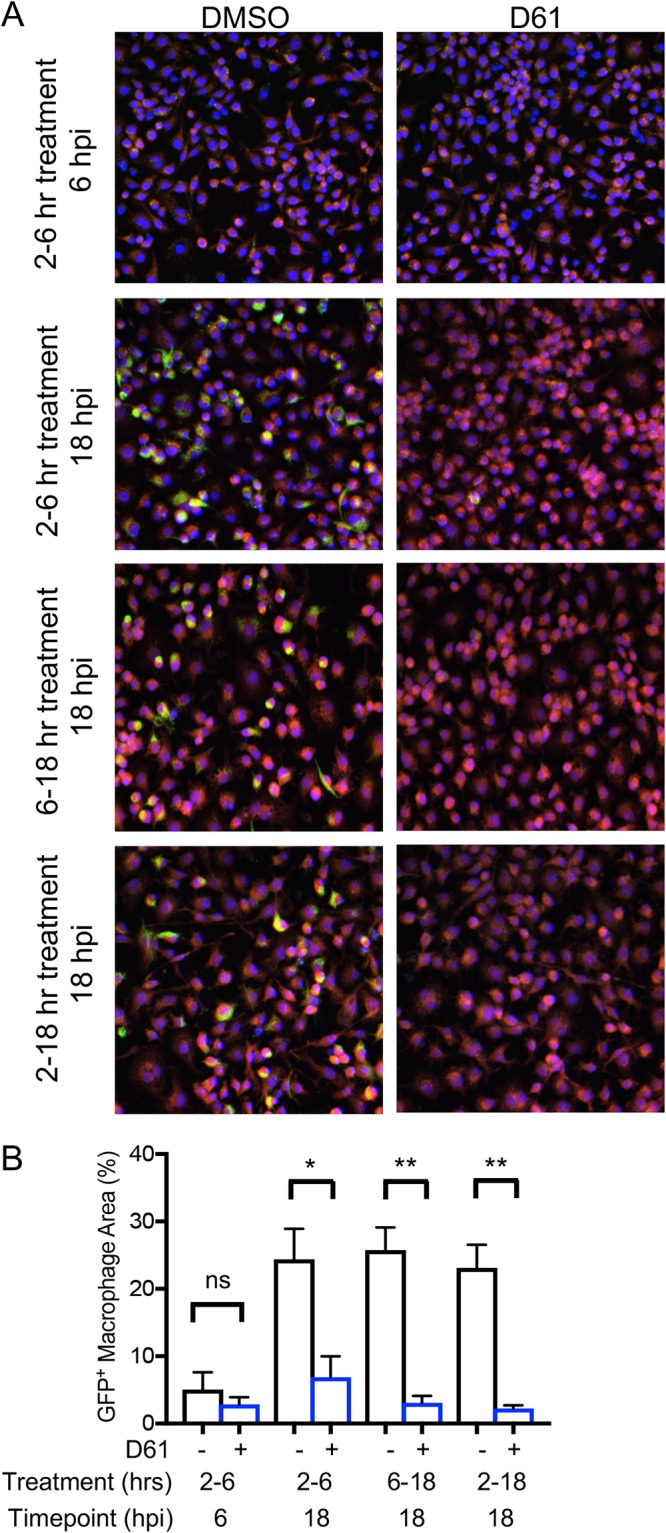
D61 suppresses *Salmonella* over the course of macrophage infection. (A) RAW 264.7 macrophage-like cells were infected with stationary-phase *Salmonella* harboring a chromosomal *sifB*::GFP insertion. Cells were treated with vehicle control or D61 (25 μM) at the indicated time points. (B) At 6 or 18 h, the cells were processed according to the SAFIRE protocol. The data are means and SEM from four independent biological replicates. *, *P* ≤ 0.05; **, *P* ≤ 0.005 (unpaired *t* test); ns, not significant.

Macrophages produce more and varied kinds of antimicrobial defenses as infection progresses and depend upon transcription and translation to do so. To establish when and for how long D61 must be present to inhibit *Salmonella* in macrophages, we treated cells from 2 to 6, from 6 to 18, or from 2 to 18 h after infection and monitored the bacterial load at 6 or 18 h. The micrographs show, as expected, a decline in the density of macrophages observed by DAPI (4′,6′-diamidino-2-phenylindole) staining at 6 versus 18 h in all samples, confirming that infection reduces cell number (Fig. 3A). Quantification of the GFP signal per macrophage area revealed that the levels of bacterial replication at 6 h postinfection were consistent with time-lapse microscopy data, with or without D61 (Fig. 1E and 3B). All three D61 treatment time frames yielded similar, low levels of GFP signal at 18 h postinfection, including when treatment occurs well after SCVs are established (6 h postinfection). These results suggest that D61 exerts an antibacterial effect within hours and continues to suppress bacterial replication and/or survival over the entire 16-h incubation period. D61 may therefore interfere with a complex and/or sustained macrophage defense mechanism.

### LC3 and LC3II accumulate in macrophages in response to D61 treatment.

**FIG 4:**
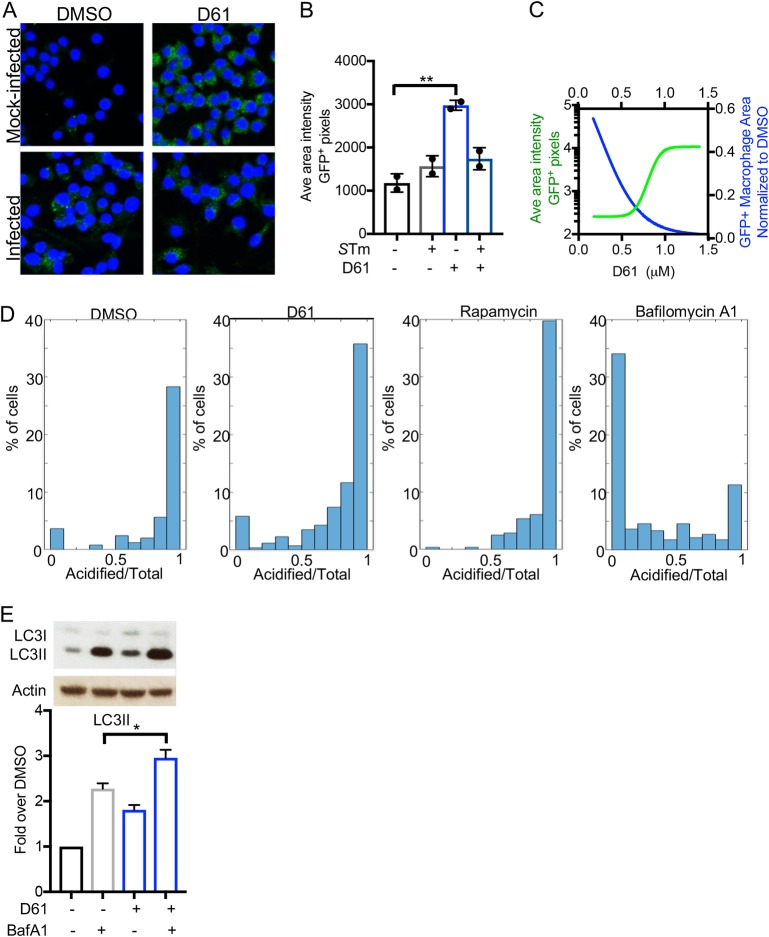
D61 induces autophagic flux through to acidification of the autophagolysosome. (A) Representative images of RAW 264.7 macrophage-like cells that were mock infected or infected with stationary-phase *Salmonella* and treated at 2 h postinfection with vehicle or D61 (25 μM). The cells were stained with DAPI (blue) and immunostained for LC3 (green). (B) The images from panel A were quantified for the intensity of LC3 signal using MATLAB SAFIRE scripts. **, *P* ≤ 0.005 (compared to uninfected, DMSO-treated cells by one-way ANOVA with Dunnett’s multiple-comparison test). (C) Macrophages were treated with 2-fold dilutions of D61 from 25 μM for 18 h and processed and quantified as in panels A and B (green line) and plotted against the data from Fig. 1D (blue line, GFP^+^ macrophage area). (D) Macrophages stably expressing an LC3-GFP, mCherry tandem sensor were treated with DMSO, D61 (25 μM), or rapamycin (500 nM) (positive control) for 6 h. As a negative control, macrophages were treated with bafilomycin A1 (200 nM) for the last 4 h of the experiment. Cells were fixed, imaged, and quantified using MATLAB. Acidified autophagosomes were identified as GFP^–^ mCherry^+^. The data are shown as histograms of acidified versus total autophagosomes derived from macrophages treated with DMSO (*n* = 247), D61 (*n* = 257), rapamycin (*n* = 279), or bafilomycin A (*n* = 326). (E) Macrophages were treated with vehicle or D61 (25 μM). At 14 h posttreatment, vehicle or bafilomycin A1 (200 nM) was added. At 18 h posttreatment, protein was extracted, resolved using SDS-PAGE, and immunoblotted for LC3 and actin. The data are means and SEM of three independent biological replicates. *, *P* ≤ 0.05 (compared to treatment with bafilomycin A1 alone by an unpaired *t* test).

Autophagy is a host cell process that can clear bacteria, including *Salmonella*, *Shigella*, *Brucella*, Mycobacterium tuberculosis, and group A *Streptococcus* (15, 16). To establish whether D61 may boost autophagy and could thereby cause the killing of wild-type *Salmonella* in macrophages, we infected RAW 264.7 cells with stationary-phase *Salmonella*, treated the samples with D61 at 2 h postinfection, and then, after 18 h, monitored the levels of LC3, a protein that contributes to, and is a marker of, autophagosome formation (17–19). Fluorescent microscopy revealed the accumulation of total LC3 (I and II) in macrophages upon infection (Fig. 4A and B), consistent with previous observations of macrophages exposed to other Gram-negative bacteria (20, 21). Treatment of uninfected macrophages with D61 also robustly increased LC3 levels compared to treatment with vehicle alone (Fig. 4A and B). We also compared the dosages at which D61 reduced the bacterial load and increased the LC3 levels. The IC_50_ values were similar (Fig. 4C), suggesting that the ability of D61 to increase LC3 levels may be related to D61-mediated killing of *Salmonella*.

The anti-LC3 antibody that we used for microscopy recognizes both cytosolic LC3I and LC3II, the lipidated form, which attaches to the double membrane of the developing autophagosome and has differential mobility on SDS-PAGE gels. Immunoblot analysis of samples from uninfected and infected macrophages demonstrated an increase in LC3II in response to D61 treatment, whereas LC3I was not significantly affected (Fig. S4A and B). These observations together suggest that D61 promotes the accumulation of LC3II in macrophages and that LC3II accumulation correlates with *Salmonella* killing by D61.

### D61 induces autophagic flux through to acidification of the autophagosome.

LC3II and autophagosome accumulation may suggest that D61 either blocks acidification/degradation of the autophagolysosome or stimulates autophagy through to acidification of the autophagosome. To distinguish between a block in autophagy or full flux, which includes acidification, we used a dual approach. First, we used a tandem GFP-mCherry-LC3 sensor to establish the fate of LC3 in individual cells treated with D61. With this sensor, GFP is quenched upon acidification, whereas mCherry expression remains intact (22–24). Macrophages stably expressing the tandem sensor construct were treated with vehicle (DMSO), D61 (25 μM), or rapamycin (500 nM) for 6 h (Fig. S4C). Treatment with bafilomycin A1 (200 nM) for the last 4 h of the experiment, which is commonly used to inhibit autophagic flux by blocking acidification (24–26), served as a negative control. Macrophages were fixed, mCherry-positive puncta were identified to mark autophagosomes, and GFP signal in the identified puncta was quantified using MATLAB analysis. We plotted the number of nonacidified autophagosomes (GFP^+^ and mCherry^+^) and total autophagosomes across all images. While treatment with bafilomycin A1 increased the total number of autophagosomes (red bars), nearly all of them remained nonacidified (blue bars), indicating autophagic flux was indeed inhibited. In contrast, rapamycin, a known activator of autophagic flux (27, 28), or D61 increased the total number of autophagosomes, with only a small fraction remaining nonacidified (Fig. S4D). Live imaging of macrophages stably expressing the tandem sensor treated with D61 revealed that autophagosomes indeed acidify and degrade GFP and change from yellow to red (Fig. S4E). Examination of the fraction of acidified autophagosomes within the total autophagosome population revealed that treatment with bafilomycin A1 blocked GFP quenching and thus acidification of the autophagosomes. Rapamycin increased the frequency of acidified autophagosomes, compared to DMSO (Fig. 4D), and D61 treatment yielded a similar but less marked result. These population and single cell-based data suggest that D61 does not block acidification of the autophagosome but instead, similar to rapamycin, promotes the induction of autophagy, which includes acidification by the lysosome.

If D61 promotes autophagic flux, then cotreatment of cells with both D61 and bafilomycin A1 would be expected to increase LC3 protein levels above that of either treatment alone (17, 24). We therefore quantified total LC3 protein levels in the presence or absence of D61 and/or bafilomycin A1 by immunoblotting (17, 24). We treated uninfected macrophages with D61 (25 μM) for 14 h and/or with bafilomycin A1 (200 μM) for an additional 4 h. As expected, bafilomycin A1 or D61 alone increased LC3II by ∼2.2-fold or ∼1.8-fold, respectively (Fig. 4E). Cotreatment with bafilomycin A1 and D61 increased LC3II up to 3-fold compared to untreated samples. Together with the tandem sensor data, these results demonstrate that upon D61 treatment of macrophages, more autophagosomes form and proceed through to acidification by the lysosome at all time points examined. Therefore, D61 induces the autophagic pathway and does not block trafficking to the lysosome and subsequent acidification.

### D61 induces LC3 aggregation near *Salmonella* in macrophages.

**FIG 5:**
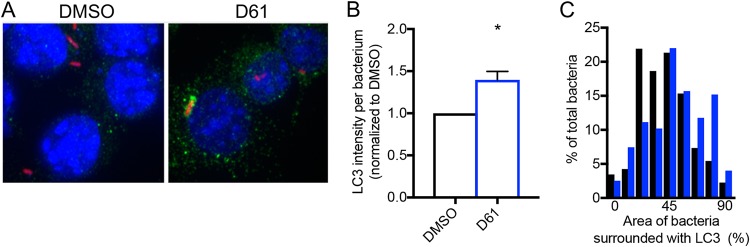
LC3 intensity increases with D61 treatment in macrophages. (A) Representative images of RAW 264.7 macrophages infected with wild-type stationary-phase *Salmonella* expressing pRFP and treated at 2 h postinfection with DMSO or D61 (25 μM). At 4 h postinfection, the cells were fixed and stained with DAPI (blue) and for LC3 (green). (B and C) Using MATLAB, macrophages within four large fields of view (4,496 × 2,761 pixels each) across four independent biological replicates were first identified. Total macrophages analyzed: WT DMSO, *n* = 296; and WT D61, *n* = 318. The bacteria were then defined as ROIs, and the LC3 intensity within a 40-pixel radius of each bacterium (red) was measured. Total bacteria analyzed: WT DMSO, *n* = 207; and WT D61, *n* = 128. (B) The average LC3 intensity per bacterium was calculated for each biological replicate. Means and SEM are shown. *, *P* ≤ 0.05 (compared to WT, DMSO-treated cells by Student *t* test). (C) Frequency distribution of the percentage of the bacterium that is positive for LC3. Black, DMSO treated; blue, D61 treated.

Prior to microbial engulfment by an autophagosome, LC3 is recruited to the vicinity of the microbe (20, 29, 30). To establish whether D61 treatment affects *Salmonella* association with LC3 in macrophages, we determined the amount of LC3 localized around individual wild-type *Salmonella*. Infected cells were fixed, stained with an anti-LC3 antibody, and monitored by immunofluorescence (Fig. 5A). Bacteria expressing RFP were identified as a region of interest (ROI) using MATLAB scripting, and total LC3 signal intensity within a 40-pixel radius of the bacterium was quantified. D61 increased LC3 signal intensity around wild-type bacteria (Fig. 5B; see also Fig. S5A). Examination of the percentage of the 40-pixel radius surrounding the bacterium that was positive for LC3 signal indicated that LC3 was more frequently found around those bacteria in macrophages treated with D61 (Fig. 5C). An average of 19.3% ± 9% of the identified bacteria were at least one half surrounded with LC3 upon DMSO treatment, whereas upon D61 treatment, an average of 38.9% ± 5% of the identified bacteria were at least one half surrounded with LC3 (Fig. 5C). Manual counts of bacteria that were fully encircled by or decorated with aggregated LC3 at their poles, which is presumably forming autophagosomes (31), revealed that with D61 treatment, 13.3% ± 6.1% of bacteria were associated with these patterns of LC3, whereas only 4.6% ± 1.6% of bacteria were associated with these patterns of LC3 during DMSO treatment. These data corroborate and expand the immunoblotting and bacterial load results (Fig. 4) by demonstrating that, within individual macrophages, D61 treatment correlates with LC3 recruitment to phagosomes and with bacterial killing.

### The antibacterial activity of D61 in macrophages requires early steps in the autophagy pathway.

**FIG 6:**
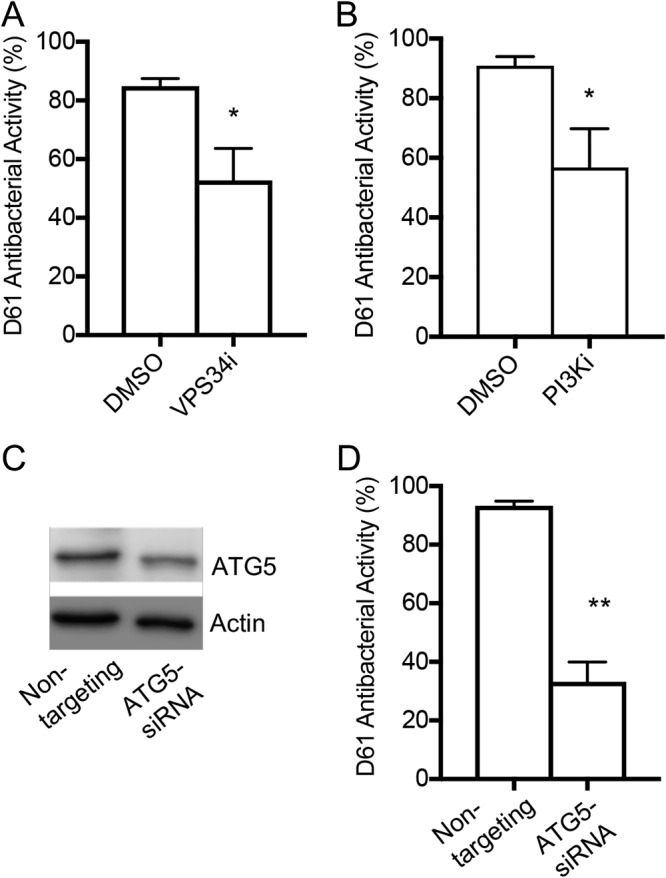
The VPS34 complex and ATG5 contribute to D61 antibacterial activity. RAW 264.7 macrophages were pretreated with VPS34 inhibitor (SAR 405) (A) or PI3K inhibitor (LY294002) (B) for 1 h. Cells were then infected with *Salmonella* harboring a chromosomal *sifB*::GFP insertion. (C and D) Macrophages harboring nontargeting or ATG5 siRNA were infected with *Salmonella sifB*::GFP. For each treatment group, the average fractional GFP area (GFP^+^ macrophage area) was determined using SAFIRE and used to calculate the antibacterial activity. Means and SEM from three to five independent biological replicates are shown. *, *P* ≤ 0.05; **, *P* ≤ 0.005 (unpaired *t* test).

To establish where in the autophagy pathway D61 may act, we sought to determine whether known chemical inhibitors of early autophagy steps reduce the antibacterial activity of D61. Phosphatidylinositol 3-kinase (PI3K) and VPS34 both play roles in the pathway prior to the recruitment of LC3II. PI3K functions at a step upstream of VPS34, a PI3K that forms the initiation complex of the pathway. To blunt autophagy, we pretreated RAW 264.7 macrophages with an inhibitor of VPS34 (VPS34i; SAR405) (32, 33) or a more generalized inhibitor of PI3K that also inhibits VPS34 (PI3Ki; LY294002) (34, 35). At 2 h after infection, we treated the samples with D61 (25 μM) and at 18 h postinfection we quantified the bacterial load based on the GFP signal. Macrophage pretreatment with VPS34i or PI3Ki decreased D61 antibacterial activity by approximately 40% (Fig. 6A and B). These results indicate that D61 relies upon early-stage autophagy to kill *Salmonella* in macrophages.

To genetically interrogate the role of autophagy in D61 antibacterial activity, we examined the effect of reducing the levels of ATG5 on D61-mediated killing of *Salmonella* in RAW 264.7 macrophages. ATG5 associates with ATG12 and is crucial for the conjugation of LC3 to autophagosomes (36). Transfection of RAW 264.7 cells with a pool of ATG5-directed small interfering RNA (siRNA) led to a 20 to 60% reduction in ATG5 levels compared to macrophages transfected with nontargeting siRNA (Fig. 6C). Quantification of the bacterial load based on the GFP signal revealed that the D61 antibacterial activity was reduced to only 30% in ATG5 knockdown cells, while it remained above 90% in control macrophages (Fig. 6D). These results confirm that D61 curbs *Salmonella* infection by inducing autophagic flux.

### D61 reduces *Salmonella* load in infected mice.

**FIG 7:**
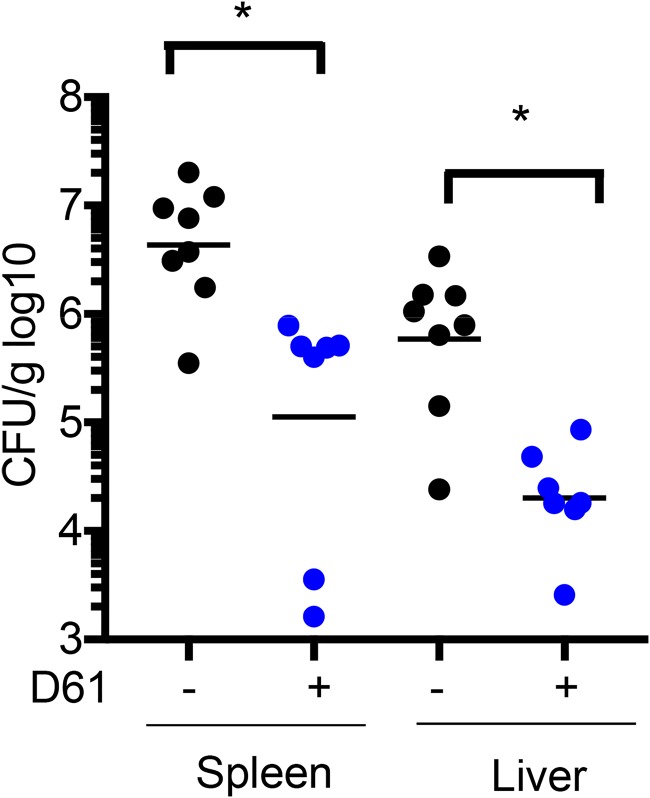
D61 reduces *Salmonella* tissue load in C57BL/6 mice. Mice were i.p. inoculated with 1 × 10^4^ WT CFU. At 20 min and 24 h postinfection, mice were dosed with 10 to 20 mg/kg D61 by i.p. injection. At 48 h postinfection, the mice were sacrificed, and the spleens and livers were immediately homogenized and plated for enumeration of CFU. *, *P* ≤ 0.05 (Mann-Whitney).

Our *in vitro* data suggest D61 is a highly potent antimicrobial agent, so we next tested whether D61 is effective in mice by comparing bacterial burden in the tissues of infected and treated mice. C57BL/6 mice were intraperitoneally inoculated with 1 × 10^4^ wild-type *Salmonella* and treated with 10 to 20 mg/kg of D61 at 20 min and 24 h postinfection. Spleens and livers were harvested at 48 h postinfection and processed for enumeration of CFU. Treatment with D61 significantly reduced *Salmonella* CFU in the spleens and livers of infected mice, indicating that this compound in particular and the promotion of autophagy in general may have therapeutic value (Fig. 7).

### Chloramphenicol and D61 synergize to reduce bacterial load in macrophages.

**FIG 8:**
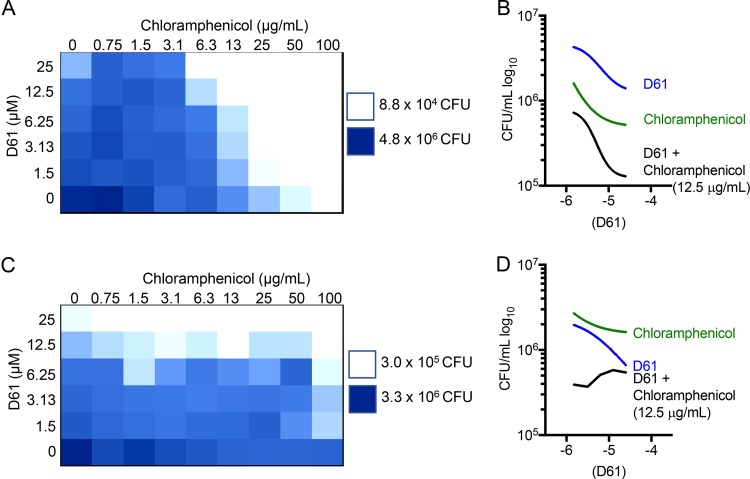
D61 potentiates the activity of chloramphenicol in primary macrophages. BMDMs were infected with stationary-phase wild-type *Salmonella* (A and B) or wild-type *Salmonella* harboring a plasmid conferring chloramphenicol resistance (C and D). At 2 h postinfection, BMDMs were treated with single or combinatorial therapy of chloramphenicol and D61. At 18 h postinfection, the cells were lysed and plated to enumerate the CFU. The results from four independent experiments were plotted on a heat map (A and C) or are presented as the CFU versus the concentration of D61 (B and D).

The antibiotic chloramphenicol inhibits the bacterial 50S ribosomal subunit and also induces autophagy in eukaryotic cells (37–41). We therefore sought to determine whether D61 may act in synergy with chloramphenicol against *Salmonella* in primary macrophages. BMDMs were infected with *Salmonella*, treated with single or dual therapy, and lysed at 18 h to enumerate CFU, as displayed in a checkerboard format (Fig. 8A). Treatment with only D61 significantly reduced bacterial load from 6.25 to 25 μM, and treatment with only chloramphenicol significantly reduced the bacterial load at all concentrations tested. However, D61 potentiated 12.5 μg/ml chloramphenicol treatment from 3.13 to 25 μM and potentiated 6.3 μg/ml chloramphenicol treatment from 12.5 to 25 μM (Fig. 8B). To determine whether synergy relied upon chloramphenicol antiribosomal activity or autophagy induction, we examined a *Salmonella* strain resistant to chloramphenicol [*Salmonella* + pYACYC184(Cm^r^)]. Chloramphenicol and D61 were synergistic only at the highest concentration of D61 (25 μM), suggesting synergy relies primarily upon chloramphenicol inhibition of bacterial protein synthesis (Fig. 8C and D). These results underscore the potential and complexity of dual treatment to increase the efficacy of antibiotic therapy.

## DISCUSSION

Omic studies have revealed many host cell changes that occur in the context of *Salmonella* infection, but it is not clear how these alterations contribute to controlling bacterial replication, nor are their roles in different cell types understood. The use of chemical genetics to identify small molecules of unknown function that alter the host-pathogen interaction is an alternative, not widely utilized approach to understanding disease and potential approaches to therapy (42, 43). Our current work describes a small molecule, D61, that is antibacterial in human and murine macrophages but not epithelial cells. We found that D61 induces autophagy to kill bacteria in macrophages.

Autophagy is a conserved cellular response to metabolic stress and to invading pathogens that all cell types examined can activate. Damaged organelles or intracellular bacteria are recognized by proteins within the autophagy pathway (e.g., p62 and LC3) and engulfed by a double membrane autophagosome, which then fuses with the lysosome for degradation of the vesicle contents. In macrophages, autophagy appears to eliminate *Salmonella* that are attenuated for virulence, including strains that cannot deploy T3SS2 (16, 44). T3SS2 is needed by *Salmonella* to block AMPK activation and thereby protect the bacteria from autophagy during the first 4 h of infection in Nramp1^−/−^ BMDMs (45). In fibroblasts, autophagy captures and removes some wild-type *Salmonella* and allows others to escape (46), but what determines the fate of an individual bacterium is not clear. Our results show that D61 curbs wild-type *Salmonella* infection in macrophages beginning within 4 h of treatment and sustains and/or increases in potency over the course of 16 h of infection. These results are similar to another small molecule, AR-12, which also reduces *Salmonella* load in macrophages and mice by inducing autophagy (47). A chemical genetics screen for compounds that prevent mycobacterial intracellular replication likewise identified an activator of autophagy, nortriptyline (42), consistent with reports that autophagy reduces mycobacterial colonization of host cells (48). These results illustrate the utility of empirical screens for dissecting host pathways that defend against intracellular pathogens.

Our observation that D61 does not interfere with *Salmonella* replication in HeLa cells supports previous reports that the autophagy pathway does not promote the killing of *Salmonella* and instead may benefit bacteria in this cell type. For example, the autophagy pathway has been implicated in the repair of SCV membranes damaged by T3SS1 and subsequent bacterial replication (49). TS331-damaged SCVs are vulnerable to recognition by LC3 and subsequent targeting for bacterial destruction early during infection of HeLa cells (50). On the other hand, it has been reported that recruitment of LC3 to the *Salmonella* SCV may facilitate bacterial access to nutrients in a manner dependent on the T3SS1 effector SopB (51). It was also recently reported that in the absence of the T3SS2 effectors SseF and SseG, autophagy kills *Salmonella* in HeLa cells (52). Our data, taken with existing studies, highlight the idea that autophagy can benefit or destroy *Salmonella* depending on the microenvironment, yielding different outcomes for different bacteria within a given cell. Additional studies at the single cell level will be needed to solve this riddle.

We also found that cotreatment with low doses of D61 and the antibiotic chloramphenicol more effectively reduced the bacterial load in macrophages than did either single therapy. Most of the impact of chloramphenicol was mediated by the effect of this antibiotic on the bacterial ribosome, as opposed to its demonstrated ability to stimulate autophagy. We speculate that *Salmonella* exposed to chloramphenicol does not respond properly to the environment of the SCV due to failure to translate virulence factors such as T3SS2 (44) and is therefore more susceptible to D61-mediated autophagy than chloramphenicol-resistant bacteria. The original indication of chloramphenicol was for typhoid fever (53), but due in part to *Salmonella* resistance, chloramphenicol is now used for treatment of ocular infections, bacterial meningitis, and *Staphylococcus* brain abscesses and is being considered as a treatment for vancomycin-resistant *Enterococcus* (54–57). However, because the side effects of chloramphenicol may be severe (58), the pursuit of combination therapies has appeal. For instance, it may be possible to cotreat with a drug that promotes autophagy and thereby lower the effective dose of a toxic antibiotic.

Our studies on D61 extend previous observations regarding the cell-type-specific roles of autophagy and its effects on *Salmonella* survival and replication in macrophages versus epithelial cells. We note that single-cell quantitative microscopy was essential for establishing that D61 is a driver of autophagy, demonstrating that stimulating autophagy is sufficient to contain *Salmonella* within these cells. Why D61 treatment alone did not kill *Salmonella* in HeLa cells remains unclear. In addition, we do not know the molecular target of D61 nor whether D61 may have off-target effects, as do many small molecules (59). Advanced characterization of D61, such as drug affinity responsive target stability (DARTS) (60), RNAi screening, or proteomics analysis of macrophages treated with D61 may lead to a more refined understanding of the mode of action and determine whether control of the bacteria is linked directly to sequestration of *Salmonella* in autophagosomes and subsequent destruction or indirectly via effects on autophagy induction and autophagosome formation elsewhere in infected cells. Nevertheless, by establishing how compounds such as D61 modulate the host to reduce bacterial load at the pathway level, we uncover molecular and even cell-type-specific interactions between the host and pathogen that may ultimately be targeted to curb infection.

## MATERIALS AND METHODS

### Bacterial strains.

For imaging experiments, macrophages were infected with Salmonella enterica serovar Typhimurium strain SL1344 (*sifB*::*gfp*) (61), and epithelial cells were infected with strain SL1344 SM022 (*rpsM*::*gfp*) (62). For chloramphenicol synergy experiments, SL1344 harbored *gfp* on pACYC184(Cm^r^). For all other experiments, strain SL1344 was used for infection. Bacteria used for stationary-phase infections were grown in Luria-Bertani broth (LB) with 30 μg/ml streptomycin (and 30 μg/ml kanamycin for GFP^+^ strains) for 18 h at 37°C with aeration. For infection with late-log-phase bacteria, overnight cultures were diluted 1:33 in LB and grown for 4 h at 37°C with aeration.

### Cell culture.

Murine macrophage-like RAW 264.7, BMDMs, and HeLa human epithelial cells were grown in Dulbecco modified Eagle medium (DMEM) high glucose (Sigma) supplemented with 10% fetal bovine serum, 2 mM l-glutamine, 1 mM sodium pyruvate, 10 mM HEPES, and 50 μM β-mercaptoethanol. Cells were maintained in a 5% CO_2_ humidified atmosphere at 37°C. Experiments using RAW 264.7 or HeLa cells were performed with cultures between passages 4 and 10. BMDMs were isolated as described previously (63). Briefly, bone marrow was flushed from femurs and tibias of 4- to 10-week-old wild-type Sv129S6/SvEvTac (SV129) mice (Taconic). Mononuclear cells were separated using Histopaque-1083 (Sigma), washed, and directly seeded into assay plates at 1 × 10^5^ cells/ml in complete medium supplemented with 35% conditioned media from 3T3 cells expressing MCSF (64). Media were refreshed 3 days later. After 1 week, the media were replaced with 200 μl of fresh media, and the cells were infected.

Human monocyte-derived macrophages were derived from primary bone marrow mononuclear cells (ATCC). Monocytes were grown in DMEM high glucose (Sigma) supplemented with 10% fetal bovine serum, 2 mM l-glutamine, 1 mM sodium pyruvate, 10 mM HEPES, and 50 μM β-mercaptoethanol containing 100 ng/ml MCSF. Cells were maintained in a 5% CO_2_ humidified atmosphere at 37°C. At day 7, macrophages were scraped and seeded to 96-well tissue culture-coated plates for further application.

### Bacterial infections for SAFIRE, CFU plating, and protein extraction.

**(i) SAFIRE.** For the SAFIRE analyses, infections were performed as described previously (5). Briefly, RAW 264.7 macrophages (5 × 10^4^ macrophages in 100 μl) were seeded in 96-well black-walled glass-bottomed plates (Brooks Automation). At 24 h postseeding, bacteria in 50 μl of phosphate-buffered saline (PBS) were added to a final concentration of 1 × 10^7^ CFU/ml, which is approximately a multiplicity of infection (MOI) of 30 bacteria to one cell. At 45 min after the bacterial addition, 50 μl of gentamicin was added to a final concentration of 40 μg/ml, which did not affect intracellular infection but inhibited the replication of extracellular bacteria. At 2 h postinfection, 1 μl of compound (D61; SEW06622; MolPort) or vehicle control was added to yield a final concentration of 25 μM. At 17.5 h postinfection, PBS containing MitoTracker Red CMXRos (Life Technologies) was added to a final concentration of 100 nM. After 30 min, 16% paraformaldehyde was added to a final concentration of 1%, followed by incubation at room temperature for 15 min. The wells were washed, stained with 1 μM DAPI, and stored in 90% glycerol in PBS until imaging. Image analysis was performed with MATLAB R2018a (University of Colorado at Boulder, license number 361635), as described previously (5).

**(ii) HeLa cells.** For HeLa cell analyses, infections with *Salmonella* were performed as described above except that 1 × 10^4^ cells were seeded (at an MOI of ca. 150 bacteria to one cell). Cells were infected with *Salmonella* constitutively expressing GFP from the *rpsM* locus [SL1344 (*rpsM*::*gfp*)]. Plates were centrifuged for 5 min at 500 × *g* after the addition of bacteria to enhance infection.

**(iii) CFU.** For CFU determinations, infections were performed as described above, except the cells were seeded in 96-well tissue culture coated plates (Greiner). At 18 h postinfection, the wells were washed three times in PBS, lysed with 30 μl of 0.1% Triton X-100, diluted, and plated to determine the CFU.

**(iv) Immunoblotting.** For the immunoblotting analyses, 1 × 10^6^ macrophages were seeded in 6-well tissue culture-coated plates in 1 ml of medium. At 24 h postseeding, bacteria in 500 μl of PBS were added to a final concentration of 1 × 10^7^ CFU/ml, which is an MOI of ∼15 bacteria to one macrophage. At 45 min after bacterial addition, 500 μl of gentamicin was added to a final concentration of 40 μg/ml, which did not affect intracellular infection but inhibited the replication of extracellular bacteria. At 2 h postinfection, 2.5 μl of compound or vehicle control was added to yield a final concentration of 25 μM. At 18 h postinfection, the wells were washed twice with PBS and scraped in 300 μl of ice-cold radioimmunoprecipitation assay buffer (10 mM Tris-HCl [pH 8.0], 1 mM EDTA, 1% Triton X-100, 0.1% sodium deoxycholate, 0.1% sodium dodecyl sulfate, 140 mM NaCl, 1 mM phenylmethylsulfonyl fluoride, 10 mM vanadate). Lysate was incubated on ice for 15 min to extract protein. Cells were centrifuged at maximum speed in a microcentrifuge. Supernatants were collected, and 5× loading buffer, which included 0.1% β-mercaptoethanol, was added, followed by boiling at 100 °C for 5 min and storage at −20°C until use. Equal protein portions were separated by SDS-PAGE and transferred to polyvinylidene difluoride membrane (Sigma). Protein levels were assessed by Western blotting with anti-LC3I/II (1:1,000; Cell Signaling Technology) or anti-actin (1:3,000; Sigma) antibodies. Primary antibodies were detected using goat anti-mouse (1:3,000) or goat anti-rabbit (1:3,000) horseradish peroxidase-conjugated secondary antibodies and visualized by using SuperSignal West Pico chemiluminescence substrate (Thermo) according to the manufacturer’s instructions on X-ray film.

### Broth antibacterial activity assays.

Overnight *Salmonella* cultures were washed three times in PBS and diluted to an optical density at 600 nm (OD_600_) of 0.01 in M9 minimal medium supplemented with 100 mM Tris (pH 7.4), 0.35% glycerol, 0.002% histidine, 10 mM MgCl_2_, and 0.1% Casamino Acids. Where indicated, media were supplemented with 5 μg/ml polymyxin B. Compound was added using a pin tool (CyBio) or manually, yielding a final concentration of no more than 1% DMSO. Plates were grown with shaking at 37°C, and the OD_600_ was monitored using a BioTek Eon incubator shaker microplate absorbance reader.

Checkerboard assays used D61 diluted 2-fold into DMSO and stored at room temperature. Mueller-Hinton broth (MHB; 30 μl) was distributed into 96-well flat-bottom plates, and gentamicin was serially diluted 1.5-fold down the plate. D61 was added, followed by 270 μl of *Salmonella* from overnight cultures that were washed three times with PBS and diluted in MHB to an OD_600_ of 0.01. Plates were incubated at 37°C for 18 h and read at OD_600_ on a plate reader (BioTek Eon or Synergy H1).

### LC3B immunofluorescence and determination of localization.

Macrophages were plated as described for SAFIRE imaging. After fixation, plates were rinsed with PBS and incubated with 1.5% BSA/PBS containing 0.1% Triton X-100 for 1 h at room temperature. Plates were incubated with rabbit polyclonal anti-LC3 antibody (Cell Signaling Technology) at 1:200 in 1.5% BSA/PBS containing 0.1% Triton X-100 overnight at 4°C. Washed slides were incubated with goat anti-rabbit Alexa Fluor 488-conjugated antibody (Invitrogen) at 1:1,000 for 1 h at room temperature. Nuclei were stained with DAPI, and wells were stored with 100 μl of 90% glycerol.

Images were acquired using a Yokogawa CellVoyager CV1000 confocal scanner system with a 100×/1.40 NA oil WD 0.13 (mm) objective and a Hamamatsu Photonics ImagEM X2 EM-CCD camera (C9100-14 high-resolution format; 1,024 × 1,024 pixels). Briefly, all images acquired included three channels, 23 z-slices, and a z-range of 15 μm. Images were acquired as large montages (4,496 × 2,761 pixels; defined as a field of view), converted to maximum-intensity projections and stitched using the CV1000 viewer software version 1.06.06 and ImageJ 1.47n. Images were imported as multidimensional (3D) tiff stacks and processed using MATLAB. This script is available at MATLAB Central and titled LC3B ROI quantification.

### LC3B immunofluorescence and determination of localization: MATLAB script.

LC3B immunofluorescence and determination of localization using the MATLAB script was carried out as follows. Briefly, the LC3B ROI quantification script analyzes high-resolution monochrome images of cells infected with bacteria within a region of interest (ROI). The code autodefines thresholds for all three channels, performs watershed segmentation for the first and third channels, and quantifies the number of bacteria and nuclei present across the entire image. Next, ROIs with a radius of 40 pixels are defined with the centers of identified bacteria acting as the centroid. Finally, the integrated density of the identified objects surrounding the bacterium is quantified for each ROI.

### Inhibitors used to determine autophagic flux.

To determine whether D61 antibacterial activity was dependent on the autophagic pathway, macrophages were pretreated with SAR405 (100 nM) or LY294002 (50 μM) 1 h prior to infection.

### Transient transfection with siRNA.

RAW 264.7 cells (2.5 × 10^4^) in 48- or 96-well plates were transiently transfected using Accell siRNA technology (Dharmacon), in accordance with the manufacturer’s instructions. Briefly, the cells were incubated with 1 μM Accell nontargeting or SMARTPool ATG5 siRNA using Accell delivery media for 96 h. Medium was then replaced with complete DMEM and used for SAFIRE, Western blot, or CFU assays.

### GFP-mCherry-LC3 tandem sensor.

Briefly, to generate tandem sensor-expressing macrophages, RAW 264.7 cells were transfected using Lipofectamine 2000 and the Addgene pBABE-puro-mCherry-EGFP-LC3B-AMP (80432) plasmid. After selection, cells were flow-sorted to isolate a subset of cells with stable and consistent expression for mCherry. Sterile 1.5 high-tolerance MatTek coverslips were placed into 6-well plates and seeded at 1 × 10^6^ cells/well in 2 ml of complete DMEM. Cells were then allowed to expand in an incubator for 24 h at 37°C and 5% CO_2_. Medium was exchanged with fresh DMEM at 23.5 h after seeding. After 30 min, compounds (DMSO [0.125%], D61 [25 μM], or rapamycin [500 nm]) were added for 6 h. Bafilomycin A1 (200 nM) was added for the last 4 h of the experiment. At 6 h after compound addition, the cells were washed twice with 1× PBS and 5 μM HEPES buffered at a pH of 7.4 and then fixed in 3.7% paraformaldehyde for 20 min in the dark. The cells were then washed twice with 1× PBS–5 μM HEPES buffered at a pH of 7.4, stained with 1 μM DAPI, washed twice with 1× PBS–5 μM HEPES buffered at a pH of 7.4, and fixed using 18 μl of Invitrogen Prolong glass antifade mounting medium. Samples were then placed on a flat surface in the dark for 2 days to allow the medium to reach the peak refractive index. Samples were imaged using a Yokogawa CellVoyager CV1000 confocal scanner system with a 100×/1.40 NA oil WD 0.13 (mm) objective and a Hamamatsu Photonics | ImagEM X2 EM-CCD camera (C9100-14 high-resolution format; 1,024 × 1,024 pixels). Images were acquired as volumes with the z dimensions range set to 12 μM and a step size of 300 nm. All volumes contained a DAPI, EGFP, and mCherry channel. Finally, all RAW 264.7 cells expressing the pBABE-puro-mCherry-EGFP-LC3B-AMP plasmid were analyzed using MATLAB. This script is available at MATLAB Central and titled LC3 tandem puncta quantification.

### GFP-mCherry-LC3 tandem sensor: MATLAB script.

The LC3 tandem puncta quantification script is designed to quantify puncta in high-resolution volumes on the single cell level. The code reads in 16-bit images in the form of multidimensional 4D Tiff stacks. Briefly, the code takes each volume independently, splits the channels into subvolumes, and finally undergoes a 3D controlled watershed to obtain single cell volumes which are isolated for analysis. Each single cell volume is analyzed for the number of EGFP and mCherry puncta (note that the user must set a threshold for EGFP puncta only and that the code will adapt this threshold to accommodate for differences between EGFP and mCherry quantum yields). This threshold should be adjusted for each cell line using the negative control average endogenous EGFP puncta signal. The results for each image are exported as a dot structure with the original volumes file path, filename, and the analyzed single cell volume results for the EGFP and mCherry puncta counts for each cell. Acidified autophagosomes were defined as GFP^–^ mCherry^+^ LC3, and nonacidified autophagosomes were defined as GFP^+^ mCherry^+^ LC3.

### Infection and treatment of mice.

This study was carried out in accordance with the recommendations in the *Guide for the Care and Use of Laboratory Animals* of the National Institutes of Health (65), and all protocols were approved by the University of Colorado Institutional Committees for Biosafety and for Animal Care and Use. Seven- to eight-week-old C57BL/6 mice were intraperitoneally (i.p.) inoculated with 10^4^ CFU, and the infectious dose was verified by plating for CFU. Mice were i.p. treated with vehicle (70% DMSO) or 10 to 20 mg/kg of D61 at 20 min and 24 h postinfection. At 48 h postinoculation, infected animals were euthanized by CO_2_ asphyxiation, followed by cervical dislocation. Spleens and livers were collected, homogenized in 1 ml of PBS, and then serially diluted for plating to enumerate the CFU. Dosages were determined based on LDH toxicity and SAFIRE IC_50_ assays in primary macrophages, according to approved IACUC protocols.

### Statistics.

Each data set was analyzed for outliers and Gaussian distribution. Statistical tests were applied as indicated in the figure legends using GraphPad Prism or MATLAB. For experiments where fewer than three biological replicates were performed, or technical replicates are shown, each experiment is represented as a single dot in a scatter plot with a bar graph overlay. Bar graphs with means and standard errors of the mean are shown for all experiments with three or more biological replicates.

## Supplementary Material

Supplemental file 1

Supplemental file 2

Supplemental file 3
