# Supplementary material for: Autophagy Induction by a Small Molecule Inhibits Salmonella Survival in Macrophages and Mice
Source: Antimicrob Agents Chemother. 2019 Nov 21;63(12):e01536-19. doi: 10.1128/AAC.01536-19 (PMC6879225; doi:10.1128/AAC.01536-19)
Supplement: Supplemental file 1 [file AAC.01536-19-s0001.pdf]

Figure S1

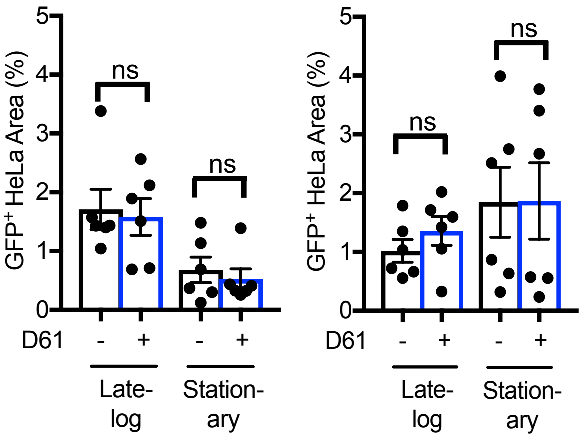

Figure S1. D61 is not antibacterial in HeLa cells infected with late-log or stationary phase *Salmonella*. HeLa cells were infected with *Salmonella* harboring GFP on the pACYC184 plasmid and grown to late-log or stationary phase, as indicated. Cells were treated at two hours post-infection with vehicle (DMSO) or D61 (25 µM). At 18 hours post-infection cells were fixed and imaged according to the SAFIRE protocol and GFP^+ Macrophage Area was quantified. Mean and SD of six technical replicates from two independent experiments.

Figure S2

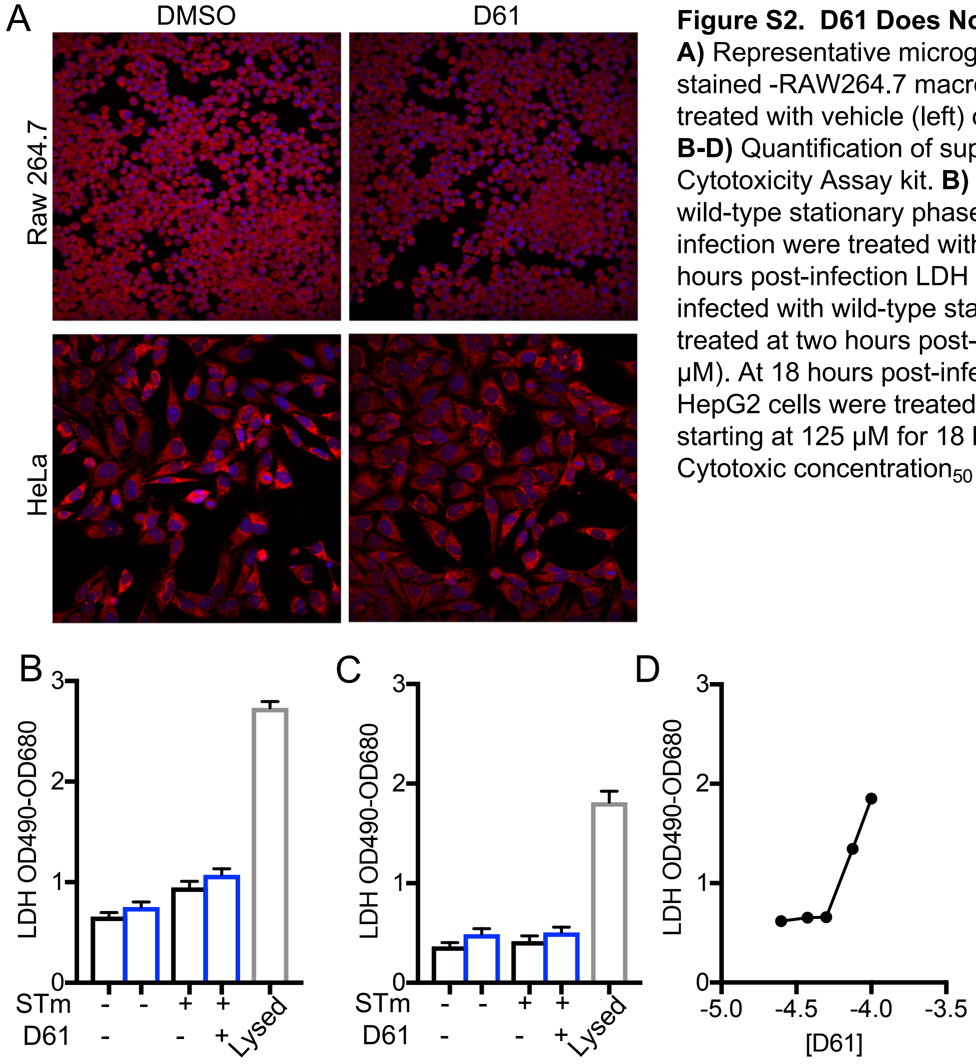

Figure S2. D61 Does Not Induce Cell Death
A) Representative micrographs from DAPI- and MitoTracker stained -RAW264.7 macrophage-like cells and HeLa cells treated with vehicle (left) or D61 ((25 μM), right) for 18 hours. **B-D)** Quantification of supernatant LDH using the Pierce LDH Cytotoxicity Assay kit. **B)** Macrophages were infected with wild-type stationary phase *Salmonella* and at two hours post-infection were treated with DMSO or D61 (25 μM). At 18 hours post-infection LDH was quantified. **C)** HeLa cells were infected with wild-type stationary phase *Salmonella* and treated at two hours post-infection with DMSO or D61 (25 μM). At 18 hours post-infection, LDH was quantified. **D)** HepG2 cells were treated with a two-fold dose-curve of D61 starting at 125 μM for 18 hours, when LDH was quantified. Cytotoxic concentration$_{50}$ (CC_{50}) = 112.5 μM.

Figure S3

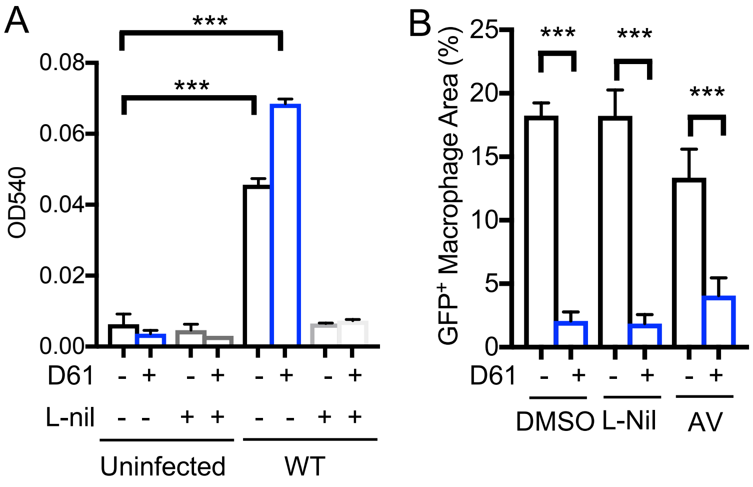

Figure S3. Nitric oxide and reactive oxygen species production in macrophages during infection does not contribute to D61 antimicrobial activity. A) Macrophages were infected with wild-type stationary phase *Salmonella* and at two hours post-infection were treated with DMSO or D61 (25 μM). At 18 hours post-infection, supernatants were collected and nitric oxide signal determined at OD540 using the Griess reagent. Mean and SEM from three independent biological replicates. *** $P \leq 0.0005$ compared to uninfected cells treated with vehicle by one-way ANOVA with Dunnett's multiple comparison test. **B)** Macrophages were infected with *sifB::*GFP-*Salmonella* and treated with DMSO or D61 (25 μM) in the presence of vehicle, L-nil (10 μM) or acetovanilone (500 μM). At 18 hours post-infection cells were fixed and imaged according to the SAFIRE protocol and GFP^+ Macrophage Area was quantified. Mean and SEM from three to six independent biological replicates. *** $P \leq 0.0005$, unpaired t test.

Figure S4

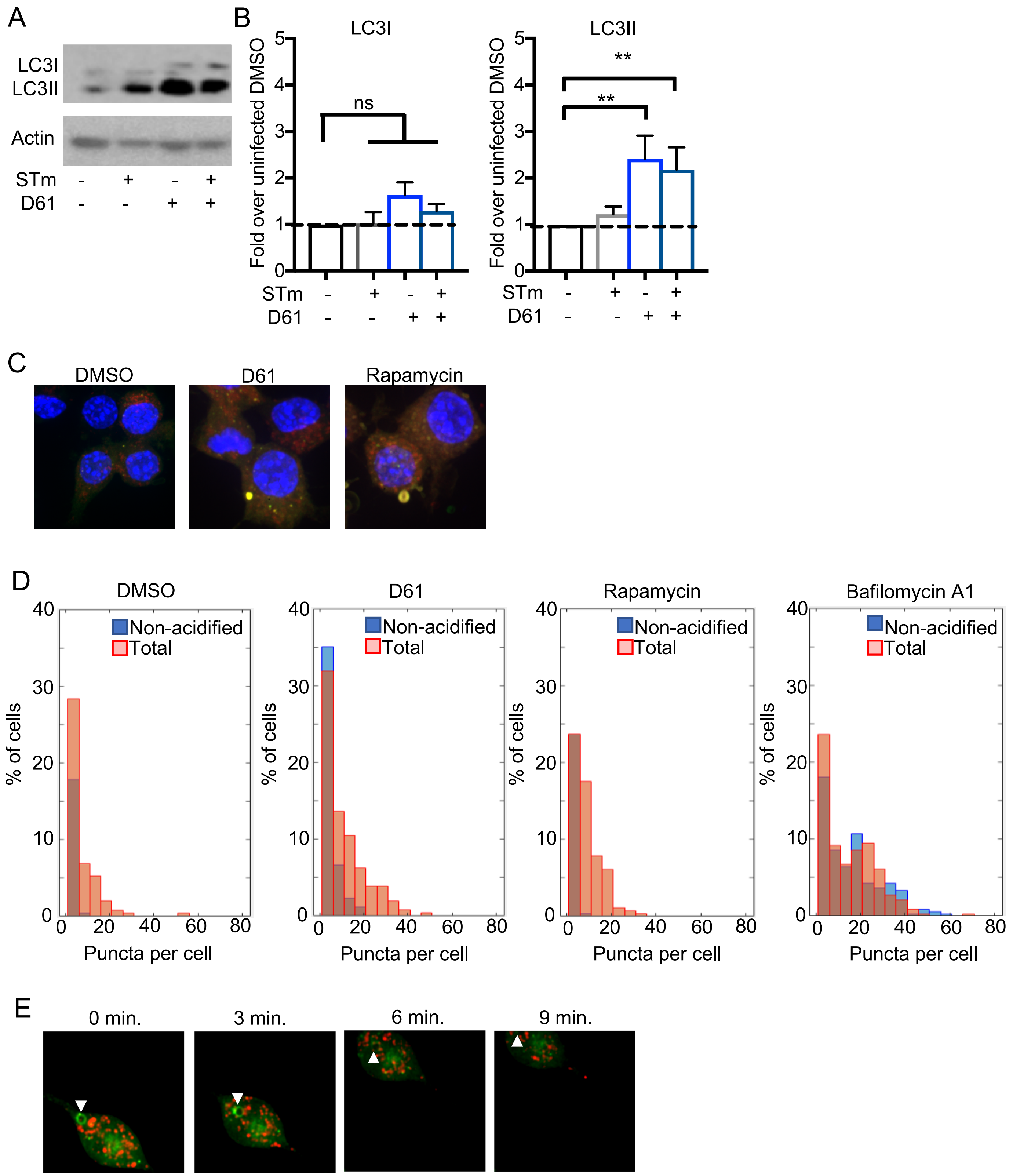
A
LC3I
LC3II
Actin
STm - + - +
D61 - - + +
B
LC3I
LC3II
Fold over uninfected DMSO
ns
**
**
STm - + - +
D61 - - + +
C
DMSO
D61
Rapamycin
D
DMSO
D61
Rapamycin
Bafilomycin A1
Non-acidified
Total
% of cells
Puncta per cell
E
0 min.
3 min.
6 min.
9 min.

Figure S4. D61 sustains autophagic flux over 18 hours. A, B) Macrophages were infected with wild-type stationary phase *Salmonella* and were treated at two hours post-infection with vehicle or D61 (25 μM); at 18 hours post-infection, protein was extracted, resolved with SDS-PAGE and immunoblotted for LC3 and actin. **A)** Representative blots are shown. **B)** Quantification of LC3I, LC3II and actin was performed using Image J. LC3I and LC3II were normalized to actin and expressed as fold change from mock-infected macrophages treated with vehicle control. Data are mean and SEM of three independent biological replicates. ** $P \leq 0.005$, compared to uninfected, DMSO-treated cells by one-way ANOVA with Dunnett's multiple comparison test; ns, not significant. **C)** Representative 100x images of macrophages stably expressing an LC3-GFP, mCherry tandem sensor treated with DMSO, D61 (25 μM) or rapamycin (500 nM) (positive control) for six hours. GFP, green; mCherry, red, DAPI, blue. **D)** Macrophages stably expressing an LC3-GFP, mCherry tandem sensor were treated with DMSO, D61 (25 μM) or rapamycin (500 nM) (positive control) for six hours; as a negative control, macrophages were treated with bafilomycin A1 (200 nM) for the last four hours of the experiment. Cells were fixed, imaged and quantified using MATLAB. Nonacidified autophagosomes were identified as GFP+, mCherry+. The normalized distribution of non-acidified (blue) and total LC3 puncta (red) across images is shown for DMSO (n = 247), D61 (n = 257), rapamycin (n = 279) or bafilomycin A (n = 326). **E)** Representative micrographs from live-imaging of macrophages stably expressing an LC3-GFP, mCherry tandem sensor treated with D61.

Figure S5

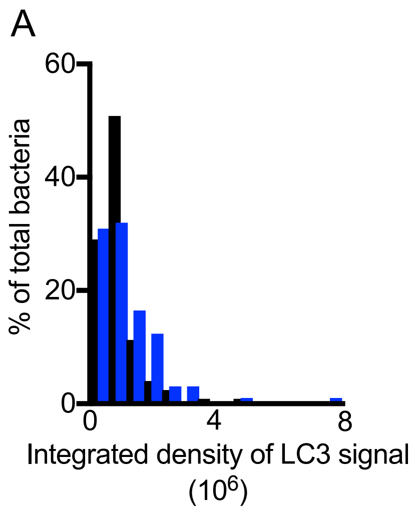

Figure S5. LC3 intensity and amount of LC3 around bacteria increases with D61 treatment in macrophages
A) RAW 264.7 macrophages were processed as described in Figure 5. The frequency distribution of the integrated density of LC3 around each bacterium is shown. DMSO-treated, black; D61-treated-blue.
